# Current Challenges in Development of Differentially Expressed and Prognostic Prostate Cancer Biomarkers

**DOI:** 10.1155/2012/640968

**Published:** 2012-08-28

**Authors:** Steven M. Lucas, Elisabeth I. Heath

**Affiliations:** ^1^Department of Urology, Barbara Ann Karmanos Cancer Institute, Wayne State University School of Medicine, Detroit, MI 48201, USA; ^2^Department of Oncology, Barbara Ann Karmanos Cancer Institute, Wayne State University School of Medicine, Detroit, MI 48201, USA

## Abstract

*Introduction*. Predicting the aggressiveness of prostate cancer at biopsy is invaluable in making treatment decisions. In this paper we review the differential expression of genes and microRNAs identified through microarray analysis as potentially useful markers for prostate cancer prognosis and discuss some of the challenges associated with their development. *Methods*. A review of the literature was conducted through Medline. Articles were identified through searches of the following terms: “prostate cancer AND differential expression”, “prostate cancer prognosis”, and “prostate cancer AND microRNAs”. *Results*. Though numerous differentially expressed genes and microRNAs were identified as possible prognostic markers, the significance of several of these genes is either debated due to conflicting results or is not validated in other study populations. A few of the articles constructed predictive nomograms using a panel of biomarkers which require further validation. Challenges to the development of useful markers include different methodology, cancer heterogeneity, and sampling error. These can be overcome by categorizing prognostic factors into particular gene pathways or by supplementing biopsy information with blood or urine-based biomarkers. *Conclusion*. Though biomarkers based on differential expression offer the potential to improve decision making concerning prostate cancer, further validation of their utility and accuracy at the biopsy level is needed.

## 1. Introduction

Prostate cancer is estimated to be diagnosed in 241,740 men in the USA in 2012 [1]. For men in developed countries, it represents the most common cancer diagnosed in men and the second most common cause of cancer-specific mortality [2]. In approximately 90% of those men who are diagnosed, prostate cancer is in a localized, potentially curable state [3]. Since PSA screening has been introduced, the mortality rate of prostate cancer has decreased in part due to improved treatment [4]. However, as evidenced by a few large randomized trials of PSA screening, prostate cancer is overtreated in many instances, subjecting patients to the morbidity of treatment [5, 6].

In an attempt to reduce treatment morbidity in patients who otherwise have indolent cancer, clinicians have developed protocols for active surveillance by which low-risk patients can be monitored for progression. However, through limitations in prostate biopsy resulting in sampling error, as many as 33% are reclassified, with the majority of these occurring on the first repeat biopsy [7]. By itself Gleason score offers the best predictive clinical feature of prostate cancer recurrence, progression, and death. Additionally, pretreatment nomograms such as the Kattan, CAPRA (cancer of the prostate risk assessment) score, and Stephenson nomograms have aided in the prediction of prostate cancer aggressiveness [8–10]. With the advent of advanced molecular biology techniques, additional information may be available to more accurately select patients that would benefit most from treatment. One of the areas in which molecular techniques would be particularly helpful is in the characterization of biopsy specimens into indolent and aggressive prostate cancers. 

In this paper, we discuss the use of differential mRNA and microRNA (miRNA) expression profiles obtained from prostate cancer tissue to improve prostate cancer prognostication. We will also discuss limitations of these techniques which include the reproducibility of the expression profiles themselves, the heterogeneous nature of prostate cancer, and the sampling error introduced by prostate biopsy. 

## 2. Gene Expression Panels

There are several different genes that have been reported to be associated with prostate cancer prognosis (Table 1). Often these genes are identified in individual studies and may have conflicting results or low sensitivities or specificities. In an attempt to increase the predictive power of these genes, a few studies have evaluated the expression of a small group of genes.

### 2.1. Small Gene Panels

Using a 4-gene panel that included PTEN, SMAD4, cyclin D1, and Spp 1, Ding et al. demonstrated strengthened association with this panel, when added to PSA [20]. The authors validated the results first in a group of 79 patients treated at Memorial Sloan Kettering, in which the C-index (a concordance index which measures the ability to discriminate between outcomes, where 0.5 is no discrimination and 1.0 is perfect discrimination) improved from 0.77 to 0.80, when adding the gene panel to the Gleason Score. In a second population derived from the Physician's Health Study (PHS), the 4-gene panel improved the C-index when added to Gleason score from 0.716 to 0.816. These 2 prior cohorts were tested using RNA expression profiles. A third validation confirmed this improvement using immunohistochemical staining of tissue microarrays of prostate cancers from 405 men treated in the PHS. 

In another model, TGF*β* and IL-7 were included in a model predicting prostate cancer survival from 44 prostate cancer specimens [15]. As a cytokine, the altered pathway of TGF*β* can stimulate angiogenesis and suppress immune infiltration in cancerous cells [36]. Conversely, decreased IL-7 activity reduces lymphocytic activity in lymphocytes [37]. Thus, in a multivariate model (which included TGF*β*, IL-7, PSA, and Gleason Score) based on radical prostatectomy specimens, both TGF*β* and IL-7 were independently predictive of prostate cancer survival, with hazard ratios of 10.4 and 0.1, respectively [15]. Furthermore, this 4-variable model nearly tripled the predictive ability of cancer survival when compared to PSA and Gleason score alone. However, it remains to be seen whether this can be duplicated in biopsy specimens. 

Using differential expression detected by microarray analysis in 102 laser capture microdissected specimens, 38 candidate genes were selected as being differentially expressed in Gleason 4 and 5 patterns versus Gleason 3 [38]. These 38 genes were used to construct a model in a case-control series of 157 high-risk patients experiencing metastasis or death in 5 years of prostatectomy versus controls that did not progress, matched for Gleason score, stage, margin status, and PSA. Through univariate and multivariate analysis a 4-variable model was constructed, which included topoisomerase-2a, cadherin 10, TMPRSS2 fusion status with ETS family transcription factors, and aneuploidy, yielding an AUC of 0.81 for the prediction of progression following prostatectomy. 

### 2.2. Larger Expression Profiles

In contrast to panels that use only a small number of genes, microarray techniques allow researchers to identify differences across a number of genes leading to larger expression profiles which contain more than 10 genes. In addition to predictive models using a small number of differentially expressed genes to assess prostate cancer prognosis, other studies have shown how larger expression profiles can also be predictive. In a study of 71 patients undergoing radical prostatectomy, Bibikova et al. used formalin-fixed, paraffin-embedded prostate cancer samples to analyze the expression profile of 512 genes [39]. A total of 11 genes were found to be positively correlated and 5 genes negatively correlated with Gleason score. Using this 16-gene panel (GEX), it was demonstrated that the GEX was more predictive of prostate cancer relapse (AUC = 0.73) versus Gleason score (AUC = 0.65). The GEX was most predictive in patients with a Gleason score of 7.

In a similar analysis by Markert et al., the expression profile of a large panel of genes was used to characterize tumors as stemlike, intermediate, or differentiated. This panel was combined with TMPRSS2-ERG fusion and PTEN status to predict the lethality of a prostate cancer [40]. Of particular importance, this expression panel was developed in 281 prostate cancers from the Swedish Watchful Waiting trial, which validates the use of the model to predict patient prognosis prior to treatment. Stem-like prostate cancers, which display a stem-cell-like prostate cancer profile along with inactivation of PTEN and p53, had the worst prognosis. ERG fusion cancers had an intermediate prognosis as did an inflammatory signature, while the differentiated group had the best prognosis. This clustering of expression profiles correlated with Gleason score, recurrence, progression, and lethality. One limitation of this model is that it was developed in a cohort of patients with a higher rate of diagnosis via TURP specimens. In order to validate this in a cohort of men primarily diagnosed on biopsy (150 men undergoing radical prostatectomy at Memorial Sloan Kettering Cancer Center), Markert et al. confirmed association of this panel of markers with Gleason score, recurrence, progression, and lethality. 

## 3. MicroRNA

Since their first description in relation to cancers in 2002 [41], the field of microRNA (miRNA) has rapidly expanded as potential biomarkers and therapeutic targets. MicroRNAs are small, functional RNA that can regulate the expression of mRNA by affecting its translation or degradation. There is thought to be 1,000 miRNAs in the human genome. Identification of miRNA targets is difficult, since typically only a small portion of the miRNA (6–8 bases) matches perfectly with the target mRNA regulatory region [42]. 

Due to their smaller size, miRNAs are very stable in formalin-fixed tissues [43]. This stability allows them to be more easily detected in prostate biopsies and serum or other fluids. Supporting this stability, Xi et al. compared the expression profiles of 40 archived colon cancer specimens collected as fresh frozen samples versus those that were collected using formalin-fixed paraffin-embedded (FFPE) samples [44]. Using locked nucleic acid microarray analysis, a strong correlation was observed (*R* ^2^ = 0.89). Furthermore, using quantitative real-time PCR, they noted that the expression of miRNA remained stable over time, despite using samples that were 10 years old. In addition to being stable during fixation, the analysis of miRNAs obtained from biopsies must be possible from the small amounts of RNA obtained from biopsy samples. Mattie et al. successfully differentiated miRNA expression in fixed prostate biopsy specimens from a premalignant and normal prostate specimen, using a linear amplification technique that allowed detection of this differential expression from a small amount of RNA [45]. Finally, Nonn et al. found comparable expression of miRNA and mRNA in fixed prostate biopsies with matched fresh frozen radical prostatectomy specimens [46].

Several miRNAs have been implicated in the initiation, progression, and metastasis of prostate cancer (Table 2). Over 150 miRNAs have been reported to be upregulated or down-regulated in prostate cancer [43]. The results of some of these studies require validation as they produce conflicting results as to whether a particular miRNA is upregulated or down-regulated, which may depend on the sample collection or study design. However, some have been reported in several studies and offer the potential to serve as prognostic markers, particularly those involved in progression and metastasis. 

### 3.1. Potential Markers Involved in Progression

MiR-21 has been found to be overexpressed in several cancers including prostate cancer [63]. In a study of apoptosis and invasiveness of prostate cancer cell lines, miR-21 was overexpressed in DU-145 and PC3 (2 less androgen-dependent and more aggressive cell lines) but not in LNCaP cell lines (a more androgen-dependent less aggressive cell line). in activation of miR-21 in the aggressive cell lines made them more susceptible to apoptosis and decreased cell motility and invasion [50]. In these cell lines, it was shown that miR-21 downregulated PDCD4, TPM1, and MARCKS, which are involved in membrane-actin interactions. Furthermore, miR-21 expression was associated with poor clinical outcomes in 169 radical prostatectomy samples [51]. Its expression was associated with stage, Gleason score, biochemical recurrence, and lymph node metastasis. In a multivariate model that included PSA, age, Gleason score, surgical margin, lymph node metastasis, and pathological stage, miR-21 was significantly associated with biochemical relapse-free survival (33.9% versus 44.5%). However, the inclusion of miR-21 in a predictive model with these variables produced only a modest improvement in AUC (0.701 to 0.714). MiR-21 may also be a suppressor of PTEN whose inhibition may lead to migration or invasion [52]. 

MiR-145 has been shown to be down-regulated in several tumor types and was associated with tumor size, grade, and prognosis [64–66]. Chen et al. identified the protein BNIP3, a repressor of the apoptosis inducing factor gene, as a target of miR-145 [67]. Using 134 FFPE prostate biopsy specimens, they noted positive expression of miR-145 in 14% of prostate cancers with progression versus 46% in cancers without progression, while the converse was true for its target BNIP3 (76.6% versus 57.1%). MiR-145 was significantly associated with disease-specific and progression-free survival on Kaplan-Meier analysis. A multivariate analysis incorporating Gleason score, PSA, and tumor stage showed that miR-145 was an independent favorable prognostic factor for progression-free survival (HR = 0.404, CI_95%_ = 0.174–0.941). 

The cluster miR-15/miR-16 has been shown to be down-regulated in prostate cancer in several studies [47–49]. Bonci et al. demonstrate downregulation of miR-15a/miR-16 in 85% of 15 prostate cancer biopsies and 20 prostate cancer cultures [48]. Increased expression of Bcl2, Ccdn1, WNT3A was noted in CaP cell lines with decreased miR-15a/mi-16. These targets are involved in cell survival, proliferation, and invasion. This microRNA cluster is a particularly attractive prognostic indicator as it is located on the chromosomal region of 13q14. While deletions of this chromosomal region are more commonly seen in metastatic CaP, a proportion of early cancers demonstrate this deletion, and the frequency of this deletion correlates with cancer progression from early to advanced to metastatic stage [68, 69]. Finally, miR15 and 16 may interact with stromal cells that support the tumor, in which their downregulation allows increased expression of fibroblast growth factor-2 and its receptor [49]. 

miR-34a is induced by p53 and can also perform many of the functions of p53 in its absence [70]. However, the absence of miR-34a impairs the activity of p53 [56]. Liu et al. demonstrated that reexpression of miR-34a can directly inhibit the growth and survival of cancer stem cells in prostate cancers, cells that are thought to be involved in progression and metastasis [57]. miR-34a and 34c may also be involved in androgen receptor-dependent, p53-mediated apoptosis and its dysregulation may be another mechanism by which cancer cells can escape androgen repression [56]. We have shown that loss of miR-34a expression in prostate cancer specimens is associated with increased expression of androgen receptor. In our study, BR-DIM (BioResponse, 3,3′-Diindolylmethane) treatment for prostate cancer resulted in the demethylation of the promoter of miR-34a, which inactivates androgen receptor [71].

### 3.2. Differential miRNA Expression in Clinical Samples

In a genomewide expression analysis of miRNAs in 60 prostate cancers compared with 16 noncancerous sections of these prostatectomy specimens, 15 miRNAs demonstrated differential expression in tumors with extraprostatic extension versus those without [54]. In contrast to other researchers, Varambally et al. miR-101 was the most consistently overexpressed in tumors with extraprostatic extension [72]. Prior research suggests that it targets EZH2, a master regulator, that silences the expression of several genes [73]. More interestingly, miR-32 was also overexpressed, and it was shown to inhibit Bim, a molecule which stimulates apoptosis [54].

In a similar study, Schaefer et al. study the mi-RNA expression profiles of samples from 76 radical prostatectomies and correlated differential expression with clinical outcome [55]. Using 24 tumors matched to 24 noncancerous samples, 15 of 470 miRNA probes studied by microarray analysis showed differential expression, which was then validated by qRT-PCR in the 76 specimens. MiR-96 correlated positively with Gleason score, whereas miR-31 and 205 demonstrated a significant negative correlation with Gleason score. The miRNAs 125b, 205, and 222 negatively correlated with tumor stage. Finally, miR96 was a predictor of recurrence-free survival, in a regression model including Gleason score and tumor stage (HR = 3.38). 

In a third study that analyzed miRNA expression profiling with prostate cancer outcome, 114 miRNA probes were used via microarray analysis to identify differentially expressed miRNAs in cancerous versus noncancerous tissue sections in 40 radical prostatectomy specimens [59]. Among the 114 miRNAs tested, 5 were downregulated in cancerous tissues relative to normal tissues (miR-23b, 100, 145, 221, and 222). 11 patients with early biochemical recurrence had 40% increase in the expression of miR-135b and miR-194, relative to 11 patients without early recurrence. MiR-135b may target the mismatch repair gene MsH2 [74], while miR-194 inhibits the de novo methyltransferase DNMT3a and methyl-binding protein involved in DNA hypermethylation (the disruption of which leads to genomic destabilization) [75]. 

## 4. Current Challenges to the Development **** of Prognostic Markers

### 4.1. Reproducibility of Prostate Biomarker Findings

Much of the difficulty in identifying prognostic markers for patients with prostate cancer stems from the conflicting findings between studies from different centers. This point is illustrated in a review article about miRNA expression profiles in prostate cancer in which the largest 5 series of miRNA expression profiling were compared [76]. In a table listing 45 miRNAs discussed in these series, 16 show discrepancies as to whether they are upregulated or down-regulated. Additionally, none of the genes are discussed as being significant through all 5 of these series. 

Directly investigating the reproducibility of gene expression profiles in different populations, Michiels et al. tested the stability of expression profiles of data from 7 publicly available expression profile studies in multiple random validation sets to predict a binary outcome [77]. Using the 50 genes most highly associated with outcome in each training set developed through microarray analysis, they found considerable misclassification across validation sets, with a minimum rate ranging from 31 to 49%. Moreover, many of the confidence intervals for these misclassification rates overlapped 50%, suggesting that in all but 2 of the studies the expression profiles were no better than chance alone in predicting outcomes. They also noted that the misclassification rate decreased with increased size of the training set. 

In order to gain more certainty with gene expression profiles obtained from microarray analysis, other methods of correlation need to be considered. Traditional methods of assessing reproducibility of gene expression profiles count the number of genes that are consistent between different datasets and express them as a percentage of the total genes expressed, termed proportion of overlapping genes, POG [78]. A recently developed metric to compare two separate gene expression panels assesses the proportion of functionally related genes that are shared between each group rather than shared expression of particular genes. This is termed proportion of overlapping genes related (POGR, or nPOGR, when it is normalized between the two groups). Though two separate datasets may have very low POG scores, their nPOGR scores can be very high. In prostate cancer, for example the consistence scores improved dramatically when changing the analysis from POG to nPOGR for the top 10 genes (0.30 to 0.89), 50 genes (0.14 to 0.69), and top 100 genes (0.15 to 0.66). This method of microarray interpretation takes into account the functional expression of a particular cancer, which is more clinically relevant than the actual genes themselves. 

Furthering this concept, Soh et al. developed a different metric for analyzing microarray analysis which takes into account the interactions between genes that may appear functionally separate [79]. This technique, termed Snet (sub network) of genes, analyzes gene expression as it relates to networks of genes that are known to interact with each other. This takes into account that certain genes are affected by other genes with a certain function, a concept which is even more biologically realistic. However, one problem with utilizing these techniques is that potential errors about the assessment of gene expression profile reproducibility may arise when the assumptions about a particular gene or gene interaction is incorrect. 

### 4.2. Overcoming Prostate Cancer Heterogeneity

Another problem with predicting outcomes of prostate cancer based on expression profiles is that many prostate cancers vary in terms of the tumor grade within the same patient. In an analysis of 115 consecutive prostate cancers, Arora et al. noted 100 (85%) of prostatectomy specimens had more than 1 focus of tumor [80]. In only 9% of cases did all the tumor foci identified reflect the overall Gleason grade. It follows, then, that one would expect heterogeneity of gene expression between each tumor foci in a single radical prostatectomy specimen. This would be particularly problematic if one assumes that multifocal prostate cancer arises from a field effect scenario, in which the separate tumors are from a polyclonal origin. However, using high-resolution genome-wide copy-number analysis, Boyd et al. suggest that even multifocal prostate cancer can have a monoclonal origin [81]. In 18 microdissected prostatectomy specimens, 13 cases were identified as having more than 1 tumor foci and thus were informative. In these cases, identical genomic copy-number changes defined by the same breakpoints were shared by all tumor foci within each individual prostate cancer case. This suggests that the multifocal prostate cancers originated from a single tumorigenic event with the resulting offspring migrating as tumor stem cells throughout the prostate tissue. Thus, at the level of these progenitor cells, there may be a high degree of consistency between tumor foci. The resulting Gleason score heterogeneity then arises from changes that occur after this migration event. If one were able to identify expression profiles common to these progenitors, these profiles would be more consistent across individual tumor profiles. However, if the later changes are more prognostically significant, the progenitor expression profiles may be irrelevant. 

Another way in which heterogeneity may be encountered with gene expression profiling is the complexity of the tissue within the prostate. Gene expression profiles based on samples obtained by crude dissection may be contaminated by neighboring normal prostate epithelial cells, prostate stroma, and infiltrating lymphocytes. In order to reduce this contamination, recent studies have used a technique of laser-captured microdissection of prostate cancer foci, which allows one to separate the malignant epithelial cells from the surrounding tissue [46]. This technique also allows researchers to examine separately the stroma adjacent to these malignant cells, which may elucidate the role of tumor-stromal interaction in prostate cancer progression. The limitation of this technique is that small amounts of RNA are extracted by this method. This needs to be amplified prior to analysis, which may introduce error into the expression profile [82]. Compared with mRNA, miRNAs require less tissue for analysis making it better suited for this technique, though both forms of RNA have been successfully obtained by this method [46]. 

### 4.3. Misclassification of Prostate Biopsies

In order for prognostic markers to guide therapeutic decisions, they must be reliably detected on biopsy specimens. However, it is known that prostate biopsies can often misclassify tumors when compared to the corresponding radical prostatectomy specimen. In a very large, recent study of Gleason upgrading, 7,643 radical prostatectomy specimens were reviewed [7]. Gleason upgrading at radical prostatectomy from Gleason 6 to a higher score occurred 36.4% of the time. Of these, 11.2% were due to a tertiary pattern, which means that there was still a 25% rate of upgrading in the primary or secondary Gleason score. 

Similar to the discrepancy incurred by using prostate biopsies to predict radical prostatectomy Gleason score, which is likely based on sampling error, one would expect misclassification of gene expression profiles obtained from biopsy cores. Nonn et al. compared expression profiles obtained from frozen tissue radical prostatectomy specimens with that obtained from paraffin-embedded prostate biopsies, using both mRNA and miRNA profiles [46]. Comparison of the mRNA expression profiles revealed that 10 of 34 (29%) differentially expressed mRNAs detected in the radical prostatectomy specimens were also detected in the prostate biopsies. Comparison of the miRNA expression profiles demonstrate improved consistency 73% of the miRNAs detected at prostatectomy were also detected in the biopsy specimens with an 8% false-positive rate. 

Another manner by which this sampling error can be overcome is by utilizing circulating or excreted expression profiles to supplement those obtained by tissue expression profiles. For instance, a panel of 5 circulating miRNAs was found to be predictive of detecting prostate cancer on prostate biopsy [83]. Another example is the use of urinary PCA3 to predict prostate cancer prognosis. Urinary PCA3 was associated with tumor volume, positive margins, tumor stage, and Gleason score [84]. 

## 5. Conclusion

The overtreatment of indolent prostate cancers as well as the potential misclassification of patients on active surveillance both warrant the development of biomarkers that can improve the prediction of prostate cancer outcome at the time of prostate biopsy. Several studies have demonstrated the utility of differential expression of mRNA and miRNA in predicting clinical outcome. These studies require validation as the findings are often inconsistent between different series. Additional limitations arise from prostate cancer heterogeneity and biopsy sampling error. Further research on these valuable prognostic tools is needed to validate their reproducibility and accuracy. 

## Figures and Tables

**Table 1:** Differentially expressed genes reported to be prognostic for prostate cancer.

	Target/function	Role in prostate cancer	Prognostic role
PTEN [11]	Regulator of PI3K pathway, cell cycle, tumor suppressor, targets miR21	Decreased invasion, migration	Gleason score, stage, BCR, metastasis

TMPRSS2-ERG [12, 13]	Trans membrane protease regulated by androgen receptor, fuses with ETS transcription factors	Increased tumor genesis, androgen independence	Diagnosis, androgen independence

Myc [14]	Transcription factor	Increased proliferation	Progression, survival, BCR after radiation

TGF-*β* [15]	Stops cell proliferation, stimulates differentiation	Increased cell growth, angiogenesis, suppress immune cells	Cancer-specific survival

IL-1 [16]	Activates NFK*β*	Increased tumor genesis	Progression

IL-6 [17]	Paracrine and anticrime growth stimulator	Increased proliferation	Progression

IL-7 [15]	Inhibits TGF*β* production	Decreased results in immune resistance	Cancer-specific survival

CCL-2 [18]	Chemokine stimulates inflammatory cell chemo taxis	Increased cell growth, invasion, metastasis	Tumor volume, Gleason score

NPY [19]	Neurotransmitter, regulates cell growth via Y1-R	Decreased cell growth, metastasis	D'Amico risk group, BCR

SMAD4 [20]	Modulates TGF*β* family of genes	Decreased differentiation, apoptosis	BCR and metastasis

Ki-67 [21]	Cell cycle regulation	Decreased proliferation	Gleason score, BCR

P53 [21]	Cell cycle regulation	Increased differentiation	Gleason score, survival

P16 [22]	Regulation of several targets (CDKN2a, TMS1)	Decreased proliferation	Gleason score

P21 [23]	Regulates cell growth	Decreased proliferation	BCR after surgery

BCL2 [24, 25]	Regulates apoptosis, affects caspases, mitochondria	Increased cell survival	BCR after surgery and radiation

BAX [26]	Proapoptotic; BCL2 gene family	Decreased cell survival	BCR after radiation

VEGF [27, 28]	Angiogenesis	Increased invasion	Gleason Score, survival, BCR

E-cadherin [29]	Intracellular adhesion in presence of calcium	Decreased invasion, migration	Gleason score, progression, survival

EZH2 [30, 31]	Targets a methyltransferase; regulates tissue inhibitors of metalloproteinase	Decreased invasion, migration	Gleason score

TRAIL [32]	Stromal TRAIL stimulates apoptosis	Decreased stromal expression-cell survival	Recurrence-free survival

PLA2G7 [33, 34]	Target of ERG, regulates actin expression	Increased migration and invasion	Gleason score, metastasis

Nuke*β* [35]	Regulates transcription, inflammation. Inversely expressed with CD10	Unregulated cell survival	BCR after prostatectomy

**Table 2:** Differentially expressed microRNAs reported to be prognostic for prostate cancer.

	Target	Role in prostate cancer	Prognostic role
MiR-15/16 [47–49]	BCL2, CCDN1, WNT3A, also fibroblast growth factor-2 in tumor stroma	Downregulated cell survival, proliferation, invasion	Indirect: 13q del progression

MiR-21 [50–52]	Inhibits PTEN, PDCD4, TPM1, MARCKS	Down regulated invasion and migration	BCR

MiR-23 [53]	Repressed by MYC	Down regulated mitochondrial activity	

miR-25 [54]	Cluster with miR-106b, inhibits PTEN	Overexpression tumorigenesis	

miR-31 [55]	Inhibits radixin	Down regulated invasion	Gleason

miR-32 [54]	Homologue of miR-25, inhibits Bim, pro-apoptotic gene	Overexpression cell survival	EPE

miR-34 [56, 57]	Target of p53	Down regulated P53 mediated apoptosis disrupted. May affect cancer stem cells	

miR-96 [55]	Inhibits hZIP1	Overexpression decrease zinc uptake	Gleason

miR-100 [47, 58]	THAP2 regulates cell cycle, SMARCA5 DNA replication, BAZ2A inhibits DNA replication		Decreased progression Increased BCR

miR-125b [55]	Inhibits BAK1	Inhibits apoptosis	Stage

miR-135b [59]	Inhibits mismatch repair gene MSH2	Overexpression genomic instability	BCR

miR-145 [60–62]	Inhibits c-MYC	Upregulated or downregulated	Gleason

miR-194 [59]	Inhibits de novo methyltransferase DNMT3a	Overexpression hypomethylation, genomic instability	BCR

miR-196a [54]	HoxB8	Overexpression cell survival	EPE

miR-205 [55]	Inhibits Laminin 5*β*3, SIP1, ZEP	Downregulation invasion	Gleason, stage

miR-221/222 [55]	Inhibit p27, p57, PTEN; stimulated by NF-*κ*B	Overexpression cell survival and proliferation	Stage
